# Whole-plant silage maize to improve fiber digestive characteristics and intestinal microbiota of Hezuo pigs

**DOI:** 10.3389/fmicb.2024.1360505

**Published:** 2024-04-25

**Authors:** Longlong Wang, Pengfei Wang, Zunqiang Yan, Pengxia Zhang, Xitong Yin, Rui Jia, Yao Li, Jiaojiao Yang, Shuangbao Gun, Qiaoli Yang

**Affiliations:** ^1^College of Animal Science and Technology, Gansu Agricultural University, Lanzhou, China; ^2^Gansu Research Center for Swine Production Engineering and Technology, Lanzhou, China; ^3^Gansu Diebu Juema Pig Science and Technology Backyard, Diebu, China

**Keywords:** Hezuo pigs, pH, whole plant silage corn, cellulase, short-chain fatty acids, intestinal microbiota

## Abstract

**Introduction:**

Utilizing roughage resources is an effective approach to alleviate the shortage of corn-soybean feed and reducing the costs in the swine industry. Hezuo pig is one group of plateau type local Tibetan pig with strong tolerance to crude feeding. Nevertheless, current research on the roughage tolerance in Hezuo pigs and the microbiological mechanisms behind it is still minimally.This study explored the impact of various ratios of whole-plant silage (WPS) maize on the pH, cellulase activity, short-chain fatty acids (SCFAs), and intestinal microbiota in Hezuo pigs.

**Methods:**

Thirty-two Hezuo pigs were randomly divided into four groups (*n* = 8). The control group received a basal diet, while experimental groups I, II, and III were given diets with incremental additions of 5%, 10%, and 15% air-dried WPS maize, respectively, for 120 days.

**Results:**

The findings revealed that compared with the control group, in Group II, the pH of cecum and colon were notably decreased (*p* < 0.05), while acid detergent fiberdigestibility, the concentration of propionic and isobutyric acid in the cecum, and the concentration of isobutyric acid in the colon were significantly increased (*p* < 0.05). Also, carboxymethyl cellulase activity in the cecum in group II of Hezuo pigs was significantly higher than that in the other three groups (*p* < 0.05). Furthermore, the cecum microbiota showed a higher diversity in the group II of Hezuo pigs than that in the control group, as shown by the Simpson and Shannon indices. Specifically, 15 and 24 bacterial species showed a significant difference in relative abundance at the family and genus levels, respectively. Correlation analyses revealed significant associations between bacterial genera and SCFAs concentrations in the cecum. The abundance of Bacteroides and NK4A214_group was positively correlated with amounts of valeric and isovaleric acid but negatively with propionic acid (*p* < 0.05). The abundance of UCG-010 was positively linked with acetic acid and negatively correlated with butyric acid (*p* < 0.05). Actinobacillus abundance was positively associated with butyric acid levels (*p* < 0.05).

**Discussion:**

In conclusion, a 10% WPS maize diet improved crude fiber digestibility by lowering cecal and colonic chyme pH, enhancing intestinal cellulase activity, improving SCFA production, and increasing intestinal microbiota diversity.

## 1 Introduction

With the improving living standards, consumers’ demand for meat, eggs, milk, and other nutritious foods has grown, leading to rapid developments in livestock and poultry farming. This also significantly increased the demand for animal feed, highlighting issues such as the shortage of feed resources and rising costs. China, a major player in hog farming and pork consumption, produced over 49 million tons of pork in 2021, accounting for 60% of the total meat consumption in the country. Thus, it is critical to efficiently develop and utilize roughage resources for reducing the costs and increasing the economical benefits of pig production.

Whole-plant silage (WPS) maize, which is nutritionally rich and high in crude fiber compared to ordinary corn forage, is a quality roughage. It has been increasingly used in grass-fed livestock production but is not yet widely adopted in pigs and other forage-feeding animals. Dietary fiber at appropriate levels can enhance intestinal microorganism fermentation in animals, leading to significant short-chain fatty acid (SCFAs) production and improving host metabolic processes (Gill et al., 2021). Studies have shown that green juicy feeds and high-quality fiber can regulate nutrient absorption and enhance immune function in pigs (De Lange et al., 2010; Knudsn et al., 2012). Wang et al. (2023) found that adding 10% WPS maize to pig diets improves feed intake and daily weight gain, and reduces pollutants and harmful gasses. Ren et al. (2021) observed that adding 80% WPS maize in Bamei pigs’ could significantly decreased the feeding costs.

Chinese local pig breeds, which have been historically grazed or fed low-nutrient-level diets with green roughage supplemented by concentrates, have developed better roughage tolerance over foreign breeds (Zhao et al., 2019). Chinese local breeds have a distinct gut flora composition and structure from imported lean-type pig breeds, which is beneficial for their stronger fiber utilization ability (Tan et al., 2020). In pigs, crude fiber digestion mainly occurs through anaerobic fermentation by microbial fiber enzymes in the large intestine (cecum and colon). Here, fiber-degrading bacteria secrete cellulase complexes, using the fiber as a fermentation substrate and producing SCFAs that are efficiently absorbed by the host (Cheng and Yan, 2008; Zhu, 2020).

The state has recently emphasized protecting and innovatively using local germplasm resources, promoting several excellent local pig breeds for industrial development. The Hezuo pig, a plateau-type Tibetan pig breed raised semi-grazing in Gansu Province’s Gannan Tibetan Autonomous Prefecture, exhibits excellent traits like good meat quality and adversity resistance. However, it has slow growth and unique nutritional requirements compared to lean pigs. It effectively digests high-fiber unconventional feeds, providing a significant advantage in utilizing China’s rural feed resources. Despite these traits, the traditional feeding method of the Hezuo pig faces challenges due to new national regulations on ecosystem protection, necessitating exploration into housed-feeding alternatives. Furthermore, there is a lack of data supporting the roughage tolerance in Hezuo pigs and the microbiological mechanisms behind it.

Our previous study found that adding different proportions of WPS maize can significantly improves growth performance, meat quality, and blood biochemical indices in Hezuo pigs (Yin et al., 2024). Based on this foundation, in this study, we further investigates the effects of WPS maize on fiber digestibility, cecum and colon pH values, fiber-digesting enzyme activity, SCFAs, and intestinal bacterial flora in Hezuo pigs. Our findings provide a reference for applying WPS maize in Hezuo pig feeding to revitalize pig farming in Tibetan area villages.

## 2 Materials and methods

### 2.1 Test animals and experimental design

Thirty-two healthy Hezuo pigs, aged 60 days, were selected after a 7-day pre-feeding period on a basal diet. These pigs had similar body weights, averaging 7.88 ± 0.81 kg. They were randomly divided into four groups (*n* = 8), maintaining an equal gender balance in each group. The control group received a basal diet, while experimental groups I, II, and III were given 5, 10, and 15% air-dried WPS maize diets, respectively, for 120 days.

### 2.2 Experimental ration composition and nutritional levels

The WPS maize was provided by a dairy plant in Yongdong Farm (118°72′ E, 35°33’ N, Gansu Province, China). Its nutrient content levels are listed in Table 1. Whole-plant silage maize was dried and crushed to a particle size of 1.65 mm and mixed according to the feed formula. The test rations were configured according to the Pig Feeding Standard (NY/T 65–2004) and the details are listed in Table 2.

**Table 1:** Conventional nutrient contents of whole silage corn (DM basis; %).

Items	Content
CP (crude protein)	11.36
EE (ether extract)	4.66
NDF (neutral detergent fiber)	41.29
ADF (acid detergent fiber)	29.29
Ash (crude ash)	35.49
Ca	2.47
P	0.32

**Table 2:** Composition and nutrient levels of experimental diets (DM basis; %).

Items	Control group	Group I	Group II	Group III
Ingredients				
Corn	69.00	66.10	63.20	60.30
Soybean meal	20.00	19.90	19.80	19.70
4% Premix^①^	4.00	4.00	4.00	4.00
Bran	7.00	5.00	3.00	1.00
whole corn silage	0.00	5.00	10.00	15.00
Nutrient levels^②^				
ME/(MJ/kg)	13.47	13.35	13.23	13.10
CP	15.89	15.74	15.60	15.46
CF	3.45	4.65	5.85	7.05
Ca	0.50	0.62	0.74	0.85
P	0.35	0.35	0.35	0.36

^①^Premix provided the following per kg diets: Fe (as ferrous sulfate) 2–7 g, Cu (as cupric oxide) 0.2–0.625 g, Zn (as zinc sulfate) 1.0–2.0 g, Mn (as manganese sulfate) 0.5–1.5 g, Se (as sodium sulfate) 3.0–10.0 mg, I 3.0–25.0 mg, VA 100–160 KIU, VD 30–80 KIU, VE 290 mg, VK 30.0–90.0 mg, VB1 25 mg, VB1 25 mg, VB6 25 mg, VB12 0.2 mg, pantothenic acid 180 mg, nicotinic acid 240 mg, folic acid 10 mg. ^②^Nutrient levels were calculated values.

### 2.3 Test animal feeding management

The experiment was conducted at Gansu Bailu Tourism Farm from June to October 2022. Pigs were fed thrice daily (08:00, 13:00, and 19:00). Feeding was regulated to ensure a slight surplus in the trough after satiation, with water available *ad libitum*. All groups were maintained under identical feeding and management conditions.

### 2.4 Sample collection and processing

Three days before the end of the experiment, fresh feces were collected from each group in the morning, midday, and evening. Daily collections from each pig were mixed and stored at −20°C. After the collection period, these samples were thawed, mixed, and sampled at 10% of the total weight. To every 100 g sample, 10 mL of 10% sulfuric acid was added. The dried feces were then crushed and stored at −20°C for fiber digestibility analysis.

At the end of the experiment, six pigs per group were randomly selected for slaughter. Their intestines were dissected to collect cecum and colon chyme in 15 mL centrifuge tubes for pH analysis. The remaining samples were stored in 5 mL sterilized EP tubes, flash-frozen in liquid nitrogen, and preserved at −80°C for analyzing fiber digestive enzyme activity, SCFAs concentration, and intestinal microbiota.

### 2.5 Measurement indicators and methods

#### 2.5.1 Fiber digestibility

The apparent digestibility of neutral detergent fiber, acid detergent fiber, and crude fiber was determined and calculated according to Feed Analysis and Feed Quality Testing Techniques (Zhang, 2007).


$$T\;\left(\%\right)=100\times \frac{A-B}{A}$$

T: The apparent digestibility of a nutrient; A: Nutrient content of the consumed diet; B: Content of the nutrient in manure.

#### 2.5.2 Cecal and colonic chyme pH

The pH of the cecum and colonic chyme was measured using a PHSI-3F type (Leici Instrument, Shanghai, China) pH meter.

#### 2.5.3 Fiber-digesting enzyme activity in the cecum and colon

The 0.5 g of cecum and colonic chyme was combined with pre-cooled 0.9% sodium chloride solution (V:W, 9:1), homogenized for 2 min in an ice-water bath, and centrifuged at 3500 rpm for 10 min at 4°C. The obtained supernatant was stored at −20°C for testing. The ELISA kits for carboxymethyl cellulase, fibrous di-glucosidase, microcrystalline cellulase, and cellulase activity assay were obtained from Jiangsu Meimian Industrial Co., Ltd., China.

#### 2.5.4 SCFAs concentrations in cecal and colonic chowders

The SCFAs content in the cecum and colon was determined by gas chromatography (GC). Briefly, the contents were thawed and 0.5 g of the cecum was weighed in a centrifuge tube. It was added with ultrapure water (W:V, 1.0:2.5), and the tube was vortexed for 3–5 min, and then centrifuged at 5,000 rpm for 10 min. Then, 0.2 mL of metaphosphoric acid deproteinizing solution with 2-ethylbutyric acid (2-EB) was added to 1 mL of the obtained supernatant. The mixture was vortexed and then centrifuged for 30 min at 10,000 rpm and 4°C. Finally, the SCFAs content was determined using an Agilent 7,890 N gas chromatograph. The column length was 30 m, the inner diameter was 0.32 mm, the membrane thickness was 0.5 μm, and the injector and detector temperatures were 260°C and 280°C, respectively. The carrier gas was nitrogen at a flow rate of 2.5 mL/min.

#### 2.5.5 Microflora of the cecum

Total bacterial DNA was extracted using the TianGen magnetic bead method DNA extraction kit (TianGen, China), following the manufacturer’s instructions. The purity and concentration of the extracted total DNA were assessed using 1% agarose gel electrophoresis. An appropriate volume of sample DNA was placed in a centrifuge tube and diluted to 1 ng/μL with sterile water. The diluted bacterial genomic DNA served as a template for amplifying the V4 hypervariable region of the 16S rRNA gene using primers (515F, 5´-GTGCCAGCMGCCGCGGTAA-3′ and 806R, 5´-GGACTACHVGGGTWTCTAAT-3′). Library construction was performed using the NEB Next^®^ Ultra™ II FS DNA PCR-free Library Prep Kit (New England Biolabs, China). The library was then quantified by Qubit and Q-PCR. The qualified library was sequenced (PE 250) using the NovaSeq 6,000 platform. Following Reads splicing and filtering, OTUs (Operational Taxonomic Units) clustering, and species annotation, valid data were subjected to species annotation and abundance analysis.

#### 2.5.6 Data analysis

One-way analysis of variance (one-way ANOVA) was conducted on the experimental data using SPSS 26.0. Multiple comparisons were made using the LSD method, and results are presented as mean ± standard deviation (Mean ± SEM).

Alpha diversity of the cecum microflora was analyzed using Qiime2 (Qiime2-2020.6). PCoA analysis was conducted with R software (Version 4.0.3). Significant species differences between groups were analyzed using LEfSe or R software. LEfSe analysis was conducted with LEfSe software with a default LDA score threshold of 4. R software’s *T*-test method was employed for analyzing significant species differences at each classification level. Spearman’s correlation analysis was used to examine the relationship between the cecal abundance of bacterial genera and SCFAs concentration in test pigs, using Origin 2022. Data with *p* < 0.05 denoted a significant difference, and those with *p* < 0.01 were considered highly significant differences.

## 3 Results

### 3.1 Effect of different additive ratios of WPS maize on fiber digestibility in Hezuo pigs

**Figure 1:**
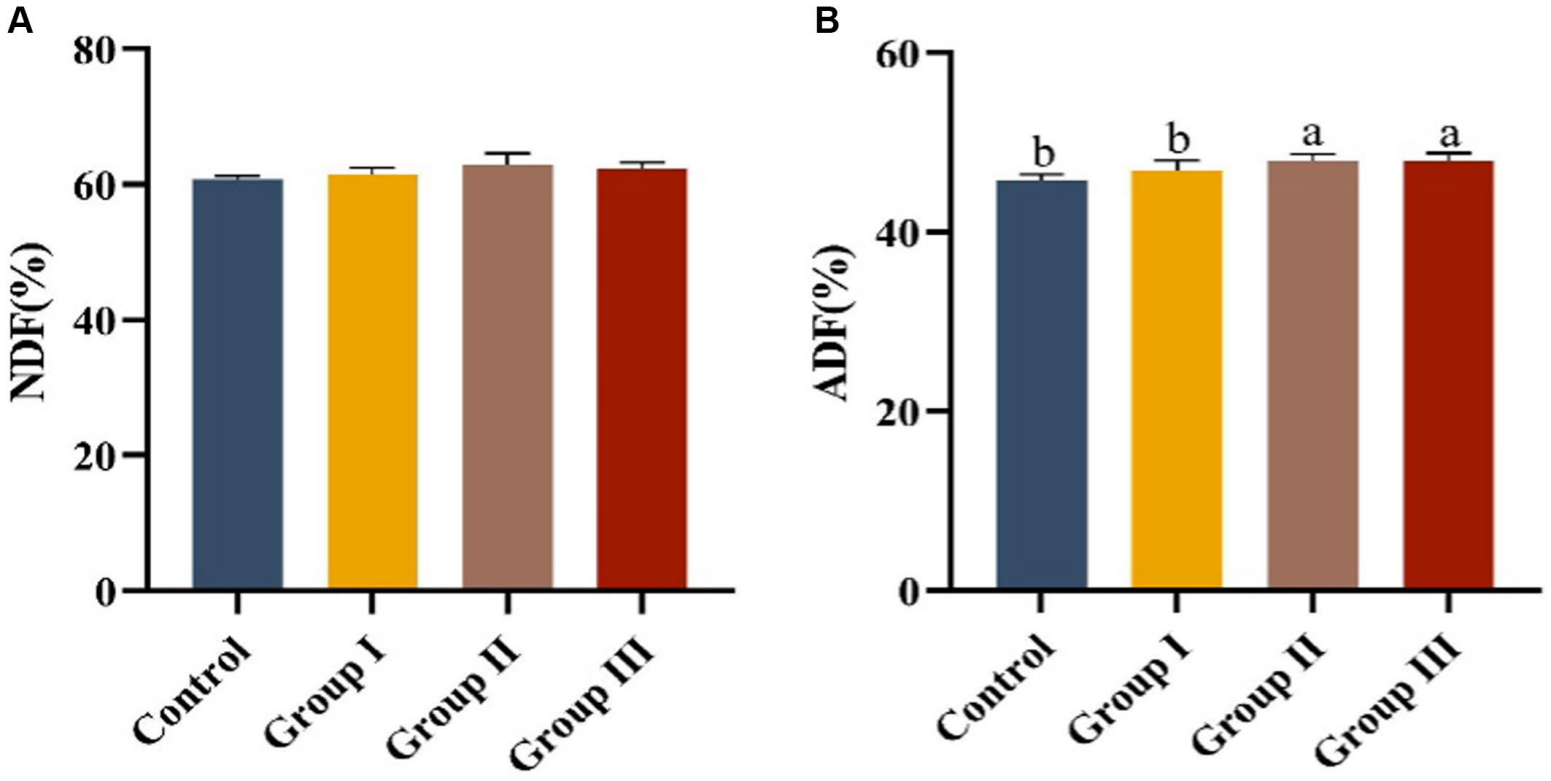
Fiber digestibility in different groups of Hezuo pigs with different additive proportions of whole plant silage maize diet. (A) NDF digestibility; (B) ADF digestibility). The same letter indicates a non-significant difference (*p* > 0.05), and different letters indicate a significant difference (*p* < 0.05); the same applies to other figures.

The effects of different additive ratios of WPS maize on the fiber digestibility of Hezuo pigs are presented in Figure 1. The differences in neutral detergent fiber digestibility among groups were not significant (*p* > 0.05). However, acid detergent fiber digestibility in group II and group III was significantly higher than in the control group and group I (*p* < 0.05).

### 3.2 Effects of different additive ratios of WPS maize on the pH of cecum and colonic chowder in Hezuo pigs

**Figure 2:**
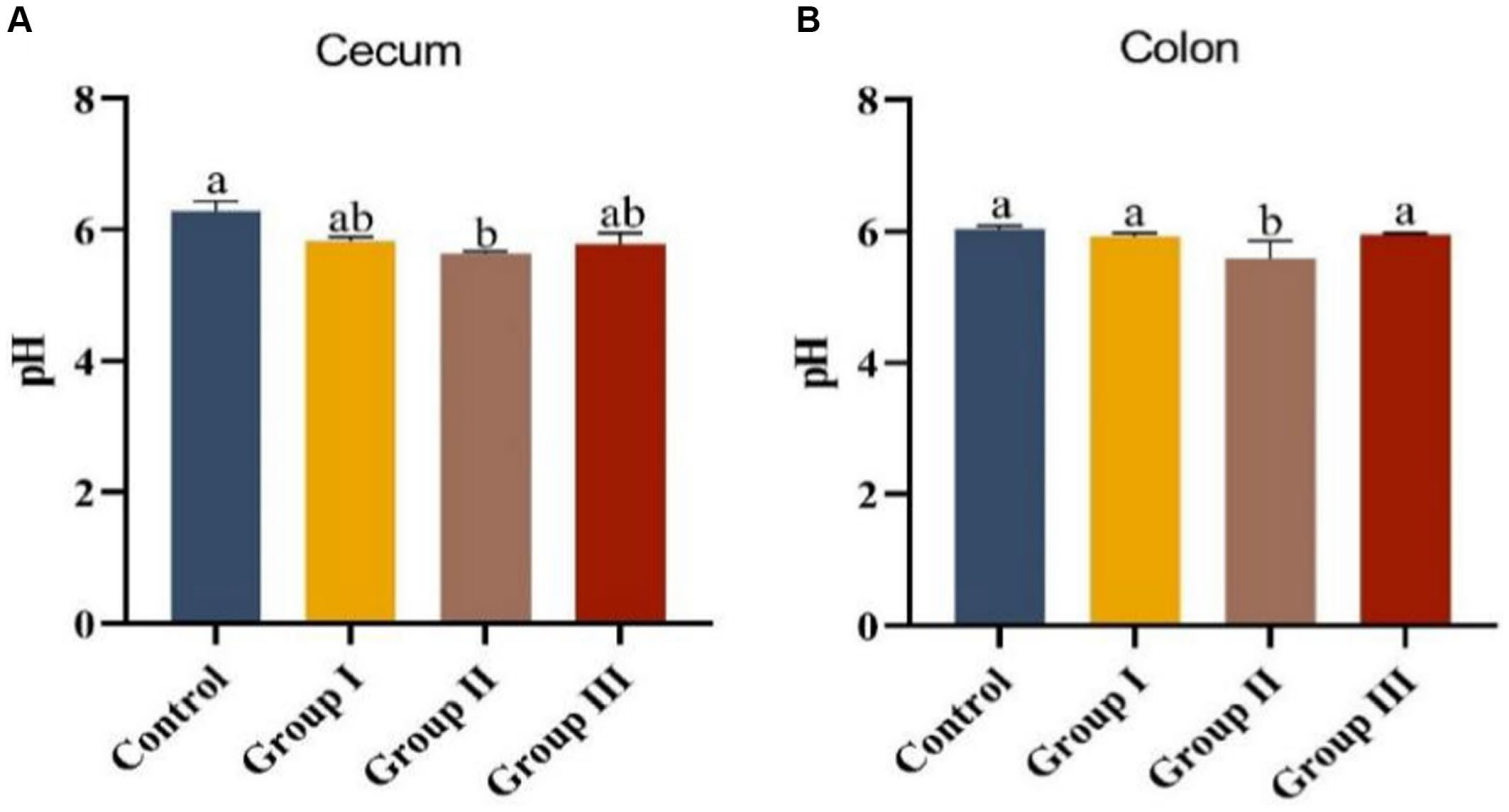
Appendix **(A)** and colonic chyme pH **(B)** in different groups of Hezuo pigs with different additive proportions of whole plant silage maize diet.

Data in Figure 2 displays the impact of varying WPS maize ratios on the pH of cecum and colonic chowders in Hezuo pigs. The pH levels in the cecum and colonic chowders of the test groups were lower compared to the control group. Notably, the colonic pH in group II was significantly lower than in the control group (*p* < 0.05). However, pH differences among other groups were not significant (*p* > 0.05). The cecum pH in group II was also significantly lower than in the other three groups (*p* < 0.05). This suggests that adding WPS maize to the diet may enhance fermentation in the cecum and colon, with a 10% addition (group II) leading to the highest fermentation level, potentially improving nutrient absorption.

### 3.3 Effects of different additive ratios of WPS maize on fiber digestive enzyme activities in the cecum and colon of Hezuo pigs

**Figure 3:**
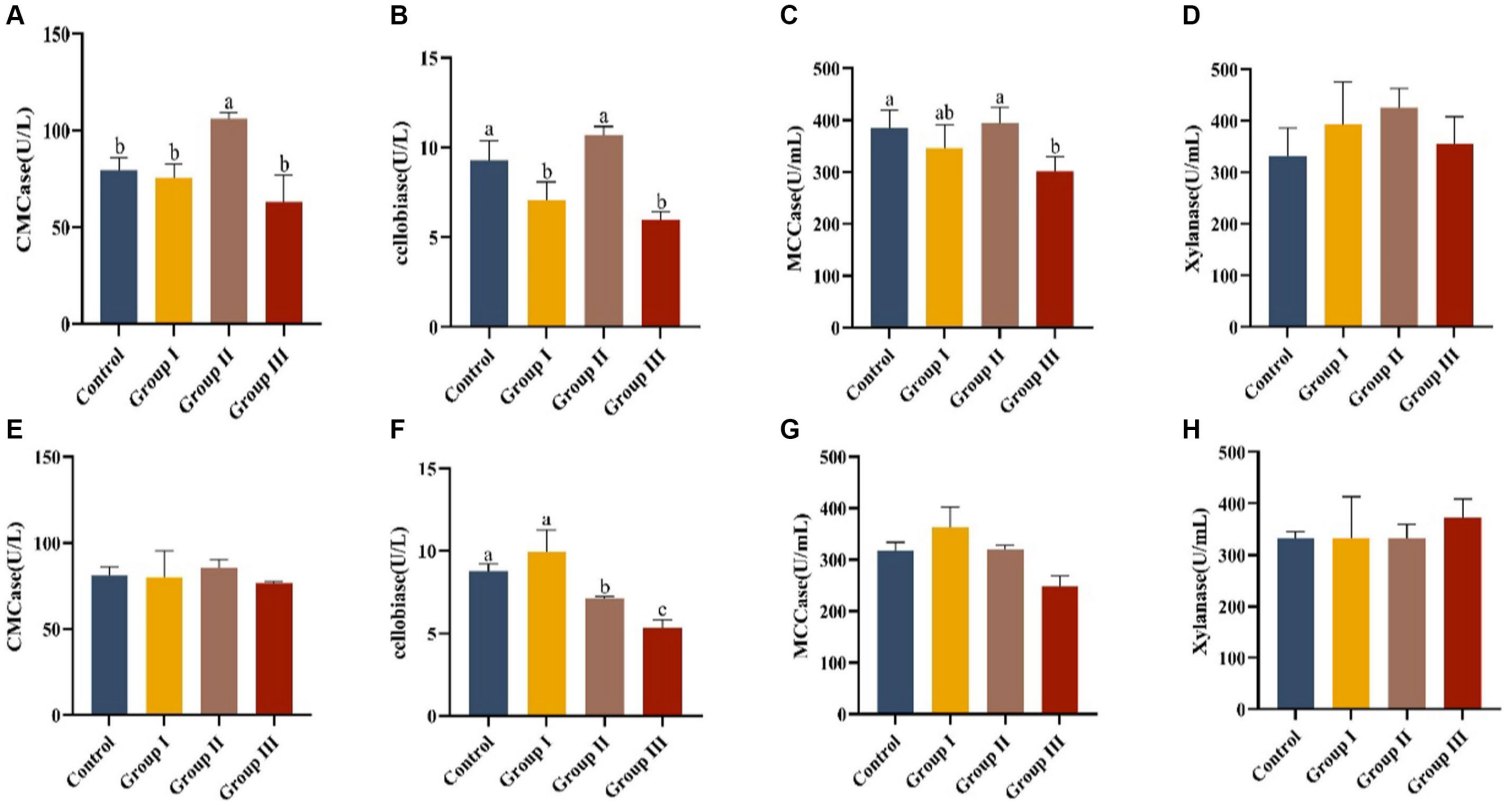
Effect of cellulase activity in the cecum **(A–D)** and colon **(E–H)** in different groups of Hezuo pigs with different additive proportions of whole plant silage maize diet. CMCase, carboxymethyl cellulase; MCCase, microcrystalline cellulase.

Data in Figure 3 presents the effects of various WPS maize ratios on fiber digestive enzyme activities in the cecum and colon of Hezuo pigs. In the cecum, carboxymethyl cellulase activity in group II pigs was significantly higher than in other groups (*p* < 0.05). Cellobiase activity was highest in group II, significantly exceeding groups I and III (*p* < 0.05). However, groups I and III had significantly lower cellobiase activity compared to the control group (*p* < 0.05). Microcrystalline cellulase activity was significantly higher in the control and group II than in group III (*p* < 0.05), with group II showing the highest activity, though not significantly different from the control group (*p* > 0.05). Xylanase activity ranked from highest to lowest as group II, control group, group I, and group III, but without significant differences (*p* > 0.05). This indicates that a 10% addition of WPS maize enhanced fiber digestive enzyme activities in the cecum.

In the colon, group I had the highest cellobiase activity, significantly differing from groups II and III, and the control group (*p* < 0.05). The activity in group III was significantly lower than in group II (*p* < 0.05). Activities of carboxymethyl cellulase, microcrystalline cellulase, and xylanase did not show significant differences among groups (*p* > 0.05). This suggests that a 5% addition (group I) of WPS maize may boost fibrous disaccharidase activity in the colon of Hezuo pigs.

### 3.4 Effect of different additive ratios of WPS corn on SCFA content in the cecum and colon of Hezuo pigs

The results of the effect of different addition ratios of WPS corn on the concentration of SCFAs in the cecum and colonic chowders of Hezuo pigs are listed in Table 3. We found the highest concentration of acetic acid and total SCFAs in the cecum of group II Hezuo pigs, which was significantly higher than in group III (*p* < 0.05); the difference between the control group, group I, and group III was not significant (*p* > 0.05). The highest concentration of propionic acid in cecal chyme occurred in group II, exceeding that of the control group and group III (*p* < 0.05); it was also significantly higher in group I than in group III (*p* < 0.05). The isobutyric acid concentration in the cecum was highest in group II and significantly higher in groups I and III compared to the control group (*p* < 0.05). Concentrations of butyric acid, isovaleric, and valeric acids in the cecum were not significantly different among all groups (*p* > 0.05).

**Table 3:** Effect of different additive ratios of whole plant silage maize on volatile fatty acids in Hezuo pigs (mmol/L).

Item	Control group	Group I	Group II	Group III	SEM	*p*-value
Cecum						
Acetic acid	24.21 ± 1.11^ab^	25.29 ± 7.63^b^	30.14 ± 0.78^a^	22.12 ± 1.49^b^	1.32	0.014
Propanoic acid	5.97 ± 0.62^bc^	8.31 ± 2.81^ab^	9.80 ± 1.18^a^	4.76 ± 0.39^c^	0.74	0.032
Isobutyric acid	0.33 ± 0.01^d^	0.54 ± 0.04^b^	0.87 ± 0.02^a^	0.47 ± 0.02^c^	0.06	<0.01
Butyric acid	2.63 ± 0.01	2.62 ± 0.02	2.76 ± 0.21	2.48 ± 0.01	0.07	0.360
Isovaleric acid	0.69 ± 0.06	0.78 ± 0.02	0.66 ± 0.02	0.66 ± 0.03	0.03	0.074
Valeric acid	0.39 ± 0.03	0.37 ± 0.02	0.38 ± 0.02	0.36 ± 0.08	0.12	0.304
Total SCFAs	33.84^ab^ ± 0.93	38.26^ab±^6.07	43.98^a^ ± 0.98	31.22^b^ ± 1.23	2.02	0.017
colon						
Acetic acid	23.03 ± 1.85	25.62 ± 0.87	27.99 ± 5.98	29.64 ± 3.48	1.66	0.328
Propanoic acid	7.84 ± 1.36	8.27 ± 2.39	7.52 ± 0.25	8.24 ± 0.19	0.7	0.259
Isobutyric acid	0.38 ± 0.04	0.44 ± 0.04	0.49 ± 0.09	0.41 ± 0.02	0.03	0.262
Butyric acid	3.68 ± 0.48	2.64 ± 0.09	3.16 ± 0.53	3.67 ± 0.22	0.21	0.107
Isovaleric acid	0.63^b^ ± 0.07	0.60^b^ ± 0.06	0.98^a^ ± 0.15	0.45^b^ ± 0.02	0.05	0.002
Valeric acid	0.71 ± 0.12	0.63 ± 0.28	0.96 ± 0.31	0.49 ± 0.16	0.11	0.218
Total SCFAs	37.12 ± 1.91	38.20 ± 1.12	40.28 ± 8.51	42.52 ± 3.76	2.19	0.355

Note: In the same row values with the same letter superscripts mean no significant difference (*p* > 0.05), while with different letter superscripts mean significant difference (*p* < 0.05).

In the colonic chyme, only the isovaleric acid concentration in group II was significantly higher than in the other groups (*p* < 0.05). The differences among the control group, group I, and group III were not significant (*p* > 0.05). Concentrations of the other five SCFAs and the total SCFA did not differ significantly among the groups (*p* > 0.05). The above results indicate that the cecum is the main site of digestion and utilization of fibrous material from WPS maize by Hezuo pigs. Adding 10% WPS maize results in the highest concentration of SCFAs in the cecum of Hezuo pigs.

### 3.5 Effect of adding WPS maize on cecal microbiota of Hezuo pigs

Based on these results, 16S rDNA sequencing was conducted on the cecum contents of Hezuo pigs in the control group and those supplemented with 10% WPS maize (group II). This was aimed to analyze the effect of the silage on the cecal microbiota.

#### 3.5.1 Diversity and PCoA analysis of cecal microbiota in Hezuo pigs

**Figure 4:**
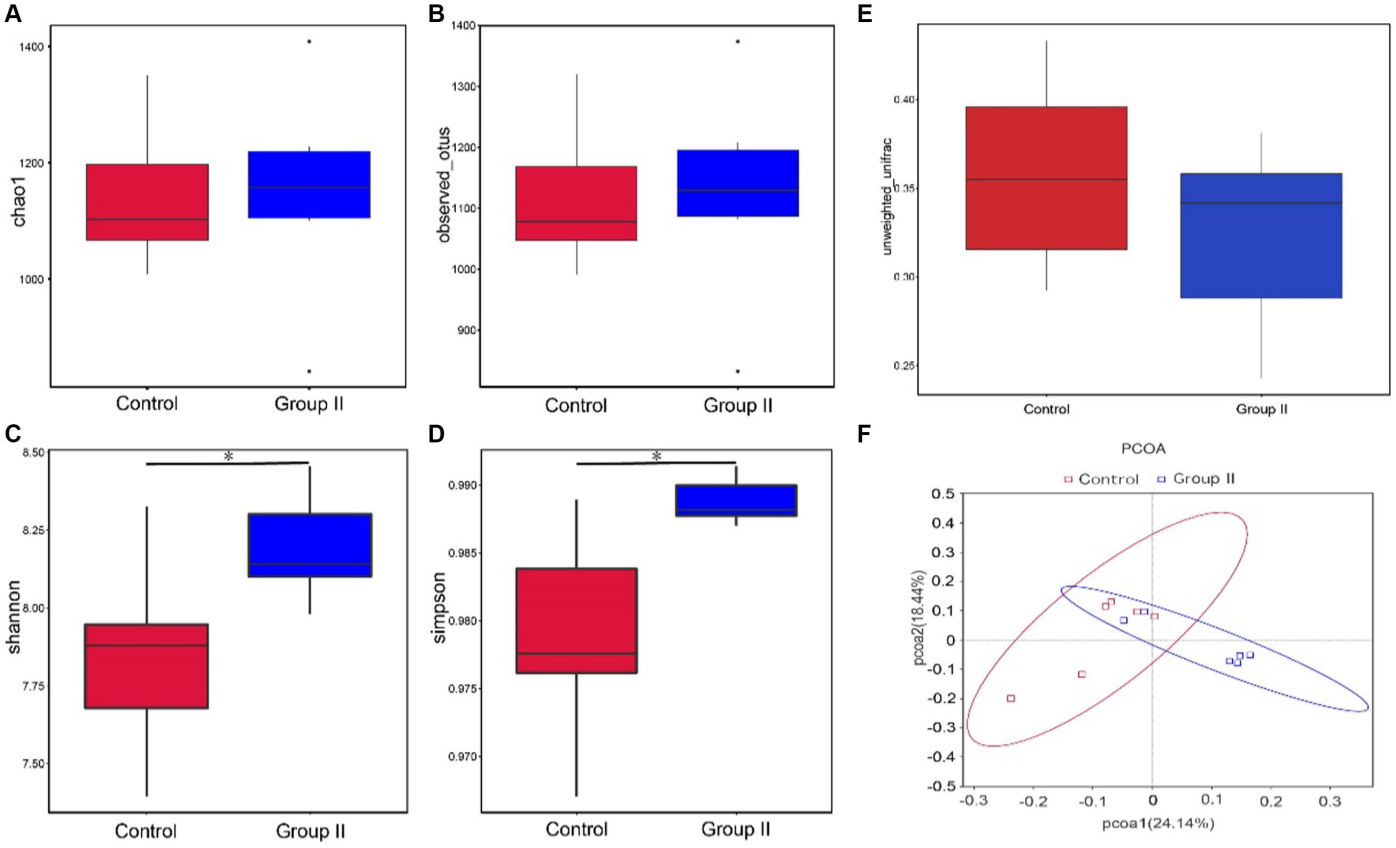
Box plots showing differences between groups in α-diversity Chao1 index **(A)**. Observed-species index **(B)**. Simpson’s index **(C)**. Shannon’s index **(D)**. β-diversity **(E)**, and PCoA analysis based on Unweighted UniFrac distances **(F)** (^*^ denotes *p* < 0.05).

The diversity analysis of the cecal microflora in group II Hezuo pigs revealed that the alpha diversity abundance indices, Chao1 and Observed-species, did not show significant differences from the control group (*p* > 0.05; Figures 4A,B). However, the diversity indices, Simpson and Shannon, were significantly higher in the silage-supplemented group (*p* < 0.05; Figures 4C,D). The β-diversity index did not differ significantly between the two groups (*p* > 0.05; Figure 4E). The principal coordinate analysis (PCoA) analysis, based on Unweighted Unifrac distance at the OUT level, indicated two principal components, PC1 and PC2, with contribution values of 34.46 and 23.40%, respectively. The plot showed a clear distinction between the cecal flora of the control and silage-supplemented groups, suggesting significant changes in the overall structure and diversity of microbial flora (Figure 4F).

#### 3.5.2 Effect of adding WPS maize on cecum intestinal microbiota composition of Hezuo pigs

**Figure 5:**
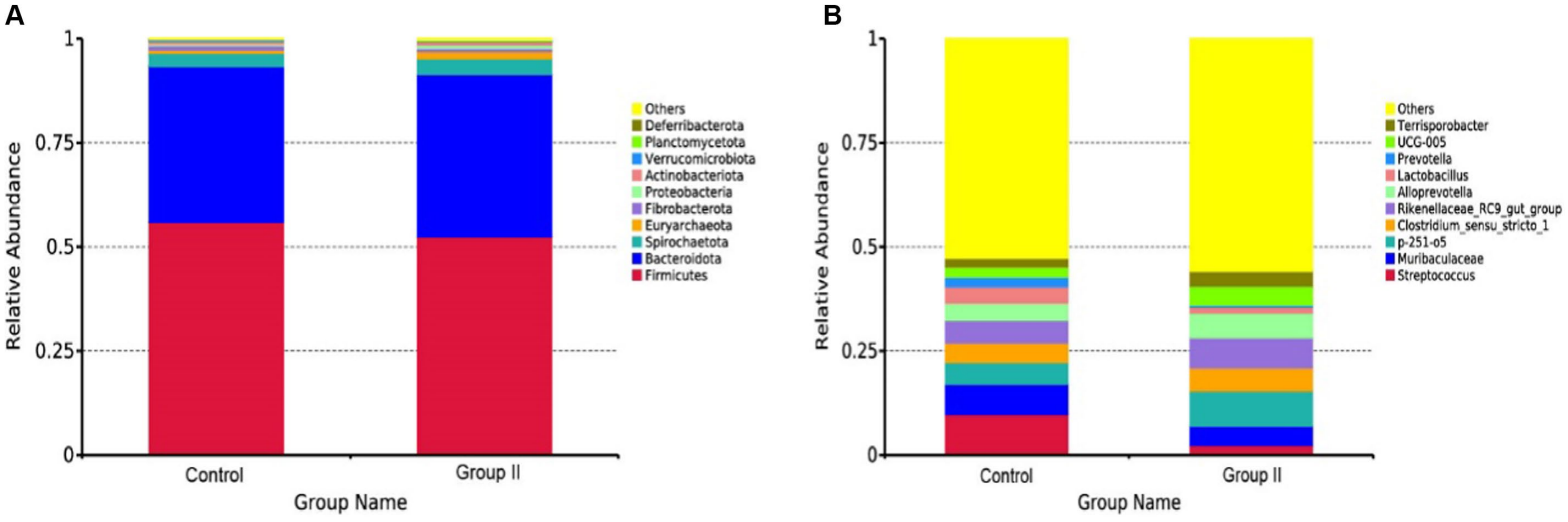
Mean relative abundance of major bacteria at the phylum **(A)** and genus **(B)** levels in the cecal colony control and 10% silage maize addition groups.

The top 10 species of Hezuo porcine cecum microflora at phylum and genus levels are presented in Figures 5A,B, respectively. The major bacterial phyla in the cecal microbiota of Hezuo pigs were *Firmicutes*, *Bacteroidota*, and *Spirochaetota* (Figure 5A), with their relative abundance comprising over 95% of the cecal microbiota in both the control and WPS corn addition groups. *Streptococcus* and *Muribaculaceae* were identified as the main dominant genera in the control group, with mean abundance values of 9.72 and 7.33% (Figure 5B). In the WPS corn addition group, *p-251-o5* and *Rikenellaceae_RC9_gut_group* were the predominant genera, with mean abundances of 8.46 and 7.28%, respectively. Relative mean abundance values of *p-251-o5*, *Clostridium_sensu_stricto_1*, *Rikenellaceae_RC9_gut_group*, *Alloprevotella*, *UCG-005*, and *Terrisporobacter* in the WPS maize addition group increased by 38.19, 16.77, 24.56, 30.21, 42.66, and 44.27% respectively, compared to the control group.

**Figure 6:**
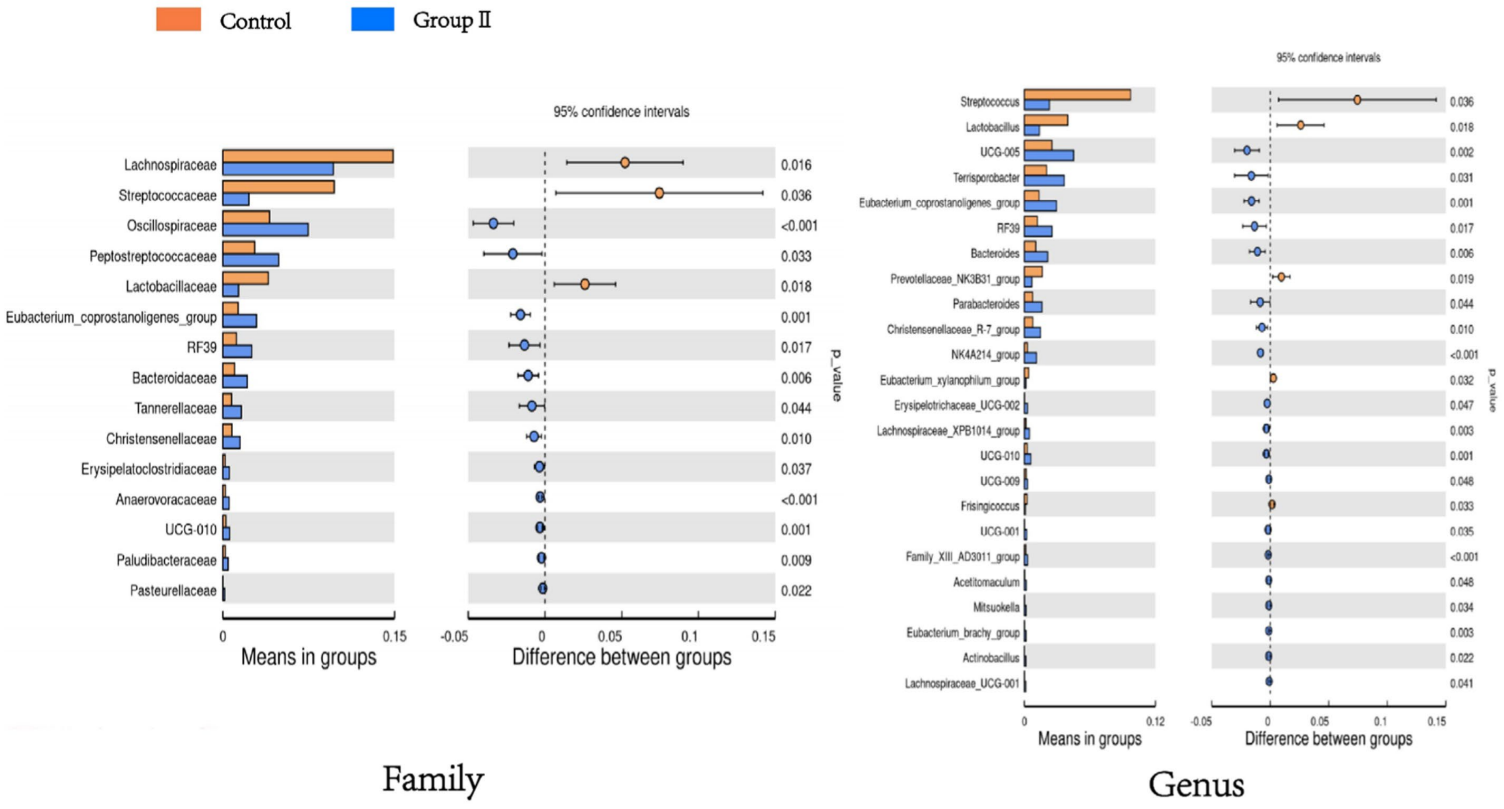
Bar graph of t-test showing differences in cecum flora between control and experimental group of pigs.

Figure 6 shows a significant difference analysis of bacterial groups annotated to the family and genus levels. At the family level, compared to the control group, the WPS corn additive group, comprising *Oscillospiraceae*, *Eptostreptococcaceae*, *Eubacterium_coprostanoligenes_group*, *Bacteroidaceae*, *RF39* etc. showed a significant increase in relative abundance (*p* < 0.05); *Lachnospiraceae*, *Streptococcaceae*, and *Lactobacillaceae* decreased significantly in relative abundance (*p* < 0.05). At the genus level, *UCG-005*, *Terrisporobacter*, *Bacteroides*, *RF39*, *Parabacteroides* etc. exhibited significant increases in relative abundance (*p* < 0.05); *Streptococcus*, *Lactobacillus*, *Prevotellaceae_NK3B31_group*, *Eubacterium_xylanophilum_group*, and *Frisingicoccus* showed a significant decrease in relative abundance (*p* < 0.05).

**Figure 7:**
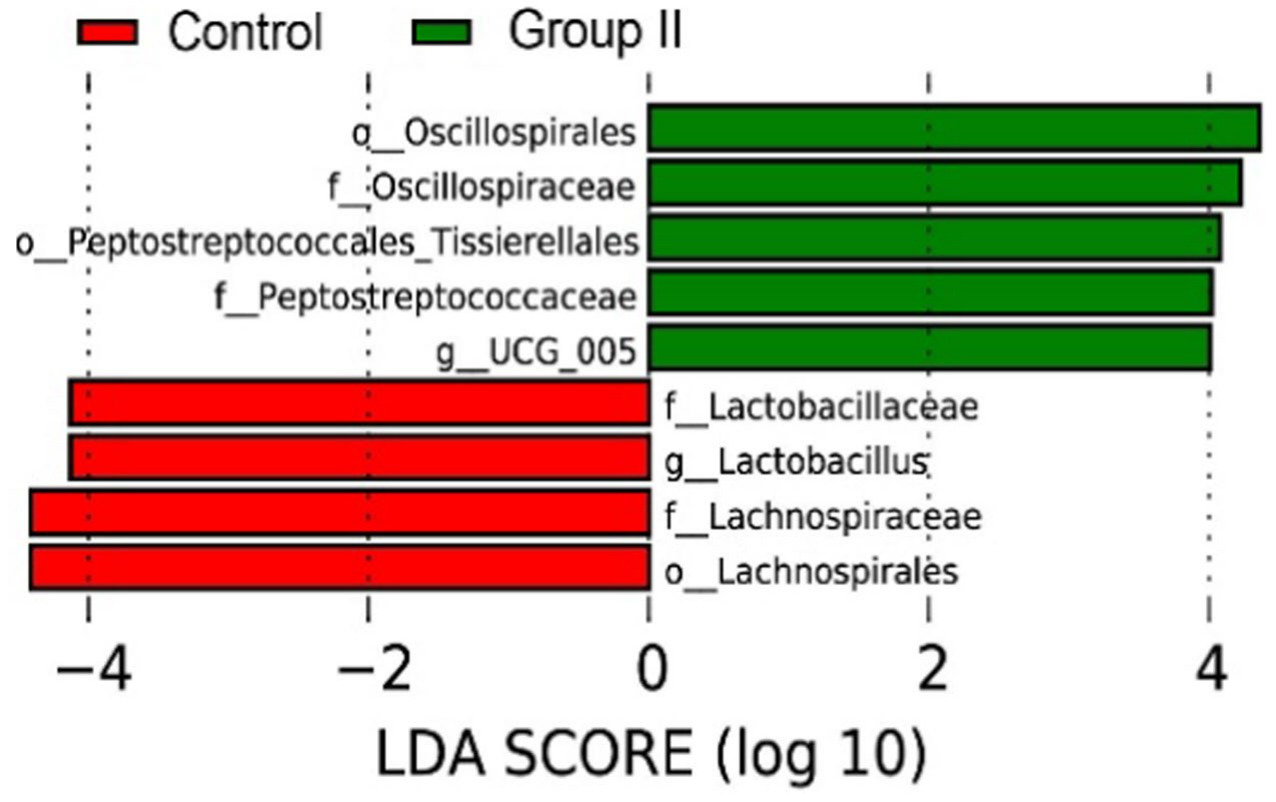
LDA bar graph for LEfSe analysis of species differences in Hezuo pig cecum microbiota.

According to the LEfSe analysis results (Figure 7), the main bacterial taxa enriched in the cecum of control Hezuo pigs were *Lactobacillaceae*, *Lactobacillus*, and *Lachnospiraceae*. In contrast, the cecum of WPS corn-supplemented Hezuo pigs showed enrichment in *Oscillospirales*, *Oscillospiraceae*, and *Peptostreptococcaceae_Tissierellales* (LDA Score > 4).

### 3.6 Correlation analysis between different bacterial genera and SCFAs

**Figure 8:**
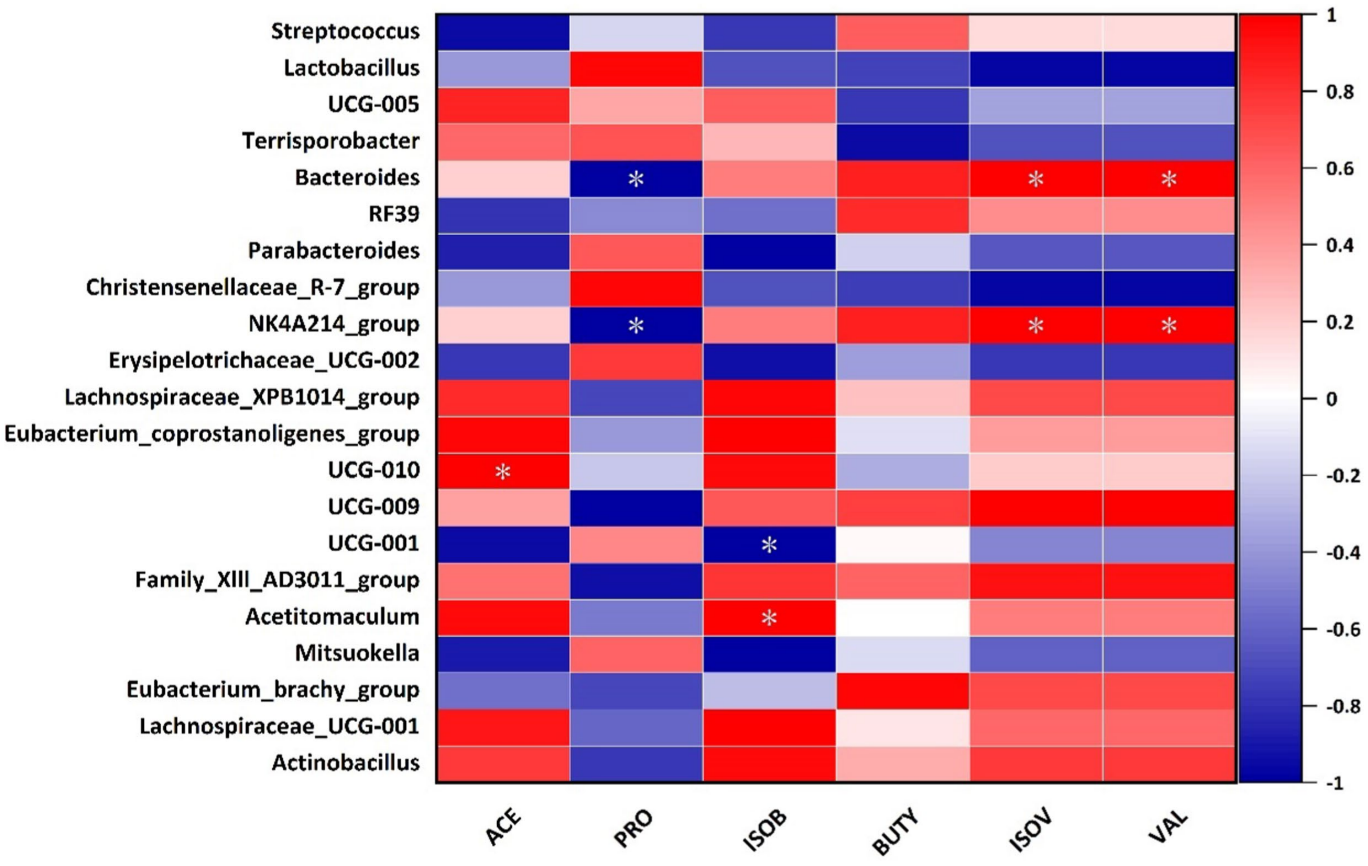
Correlation analysis between cecum intestinal microbiota and SCFAs in Hezuo pigs (^*^ indicates *p* < 0.05) (ACE, acetate; PRO, propionate; ISOB, isobutyrate; BUTY, butyrate; ISOV, isovalerate; VAL, valerate).

Spearman’s correlation analysis of SCFAs between bacterial genus levels in the cecum of Hezuo pigs in the WPS maize addition group and the control group revealed that the relative abundance of *Bacteroides* and *NK4A214_group* was significantly positively correlated with the contents of valeric and isovaleric acid (*p* < 0.05), and was significantly negatively correlated with propionic acid (Figure 8). The relative abundance of *UCG-010* was significantly positively correlated with acetate (*p* < 0.05), and negatively correlated with butyrate (*p* < 0.05). The relative abundance of *Actinobacillus* was significantly positively correlated with butyrate (*p* < 0.05).

## 4 Discussion

Dietary fiber, the feed component with indigestible plant cell wall components, cannot be digested by innate animal digestive enzymes (Peng, 1999). In pigs, the small intestine is the primary site for nutrient digestion, where most proteins, lipids, and carbohydrates are digested and absorbed. Substances that remain undigested in the small intestine, particularly fibrous materials, pass into the large intestine. Here, they are utilized by caecum and colon microorganisms (Burkey et al., 2009). Chinese local pig breeds, with their high fiber digestibility, exhibit a high tolerance to roughage (Tan et al., 2020). He et al. (2022) discovered that high-fiber diets significantly decreased the average daily feed intake in Xinhuai pigs. However, this did not negatively impact their average daily weight gain, feed-to-weight ratio, or energy utilization in the hindgut. Additionally, the diets did not adversely affect the apparent digestibility of crude proteins and neutral detergent fibers, and they moderately enhanced the apparent digestibility of crude fibers (He et al., 2022). Tan et al. (2022) observed that Tibetan pigs, under grazing conditions, demonstrate a superior ability to digest fiber, their fibrous substance digestibility exceeds that of confined-fed pigs by over 10%. Wang et al. (2023) noted that introducing 7% bran into the diet of Meishan pigs notably reduced the apparent digestibility of protein and energy, however, this did not significantly alter the digestibility of neutral detergent fiber or growth performance (Huan et al., 2020). These studies collectively indicate that local pig breeds inherently possess robust fiber digestion capabilities, and incorporating a certain amount of fiber does not detrimentally affect their growth and digestive processes. In this study, we analyzed fiber digestibility in Hezuo pigs fed WPS maize rations with varying additive ratios. We found that adding 10% WPS maize substantially increased the digestibility of acid detergent fiber by 4.92%, respectively, compared to the control group. Changes in the pH of the gastrointestinal tract directly affect the digestive and absorptive capacity of the animal intestine. An appropriate intestinal pH ensures the activity of digestive enzymes and provides a suitable environment for intestinal probiotic bacteria. This also improves the intestinal microecological balance (Luckstadt et al., 2004; Kim et al., 2005). Our results showed that the dietary addition of 10% WPS maize significantly reduced the pH of the cecum and colon of Hezuo pigs. This reduction may be because the pH of WPS maize is generally in the range of 3.9–4.2. The prolonged intake of this feed by Hezuo pigs stimulates gastric acid secretion, thus reducing the cecum pH.

Microorganisms in the digestive tract of animals produce cellulase enzymes that catalyze the breakdown of cellulose, hemicellulose, and some related polysaccharides (Li, 2019). Carboxymethyl cellulase and cellobiase aid in the digestion of cellulose, while xylanase aids in the digestion of hemicellulose (Jing et al., 2013). The level of cellulase activity is often related to the level of dietary fiber that provides the intestinal microorganisms with a fermentation substrate. Measuring the activities of microcrystalline cellulase, carboxymethyl cellulase, cellobiase, and xylanase often reflects the dietary fiber degradation ability of the animal. Varel et al. (1987) showed that increasing alfalfa fiber content (40%) in the diet of sows increased the degradation rate of xylan and cellulose, increased the number and activity of cellulolytic bacteria, and increased the number of cellulase and xylanase enzymes in pigs. This study showed that the addition of 10% WPS maize significantly increased the cecum fibrous disaccharidase activity, carboxymethyl cellulase activity, and microcrystalline cellulase activity in Hezuo pigs. It indicates that the WPS maize diet increased the relative bacterial population of certain fiber-degrading bacteria in the cecum. This increases enhanced cellulase activity and promotes the digestion and utilization of fiber in Hezuo pigs, but the mechanism still needs further research.

Most of the carbohydrates in the hindgut of monogastric animals and the rumen of ruminants are fermented by microorganisms to produce a large number of monocarboxylic acids (organic acids containing 2–4 carbon atoms and 1 carbonyl end) for the body to utilize, such as short-chain fatty acids, *β*-hydroxybutyric acid, acetoacetic acid, and lactic acid, etc. (Moschen et al., 2012). Among them, short-chain fatty acids, as important energy substances, can provide 15 to 20% of energy for monogastric animals and 75% of energy for ruminants (Bergman, 1990). In addition, short-chain fatty acids play an important role in maintaining the morphology and function of intestinal epithelial cells, intestinal microecological balance and intestinal immune response (Liu et al., 2018). Among them, acetic acid is converted into acetate, which improves the protective function of the intestinal epithelium (Chang et al., 2021). Propionic acid inhibits the occurrence of intestinal inflammation (Dupraz et al., 2021). Butyric acid is converted into butyrate, which stimulates mucin secretion in the intestinal tract and directly supplies energy to the intestinal mucosa (Gaudier et al., 2009). Hung et al. (2019) found that high levels of fiber in the diet promoted the production of SCFAs by intestinal bacteria. Carneiro et al. (2008) demonstrated that wheat fiber significantly lowered the pH of the piglet cecum and increased the cecal content of acetic and butyric acids. Pu et al. (2020) showed that feeding high-fiber diets to Suhuai pigs promoted the metabolism of SCFAs in the cecum, increasing the amount of acetic acid. Acetic acid primarily comes from cellulose and hemicellulose in plant cell walls, which are degraded by rumen’s fibrolytic bacteria (Iwamoto et al., 2001). In this study, the addition of 10% WPS maize significantly increased the cecum content of propionic acid, isobutyric acid, and total SCFAs in Hezuo pigs. It also increased the colon content of isovaleric acid, suggesting that the plant fiber of WPS maize is easily digested and utilized by the cecum of Hezuo pigs.

The intestinal flora composition is the Primary factor that influences the level of SCFAs (Dalile et al., 2019). Certain intestinal bacteria have been shown to ferment indigestible fibers into SCFAs, such as propionic acid and butyric acid. These processes mediate host energy homeostasis, immunomodulation, and mucosal barrier function (Kelly et al., 2015; Makki et al., 2018; Mendes et al., 2022). In this study, the overall structure and diversity of pig cecum flora in the WPS group changed significantly. These changes were notable compared to the control group, and the microflora alpha diversity indices, Simpson and Shannon, were significantly higher. This suggests that feeding WPS maize may promote gut health in Hezuo pigs. It does this by increasing the abundance and diversity of their gut flora and responding to changes in dietary fiber levels. Further analysis showed that at the phylum level, the major cecal bacteria of Hezuo pigs were *Firmicutes*, *Bacteroidota*, and *Spirochaetota*. Both Shoaie et al. (2013) and Greenhill (2015) showed that the intestinal flora of fattening pigs mainly consists of representatives of thick-walled *Firmicutes* and *Bacteroidota*. These findings are consistent with our results; the abundance of *Bacteroidota* increased and the abundance of *Firmicutes* decreased in the colons of Hezuo pigs fed WPS maize. *Firmicutes* and *Bacteroidota* are widely present in the intestines of herbivores. *Firmicutes* aid in the fermentation of polysaccharides in the intestines. Together with *Bacteroidota*, *Firmicutes* help hosts digest food that they cannot digest themselves. *Firmicutes* are major cellulose decomposers, whereas *Bacteroidetes* are the primary polysaccharide-degrading and utilizing bacteria. Together, they increase the utilization rate of carbohydrates, proteins, and other substances in the host. The stable balance of these two phyla plays a vital role in maintaining the micro-ecology and health of the host gut (Hooper et al., 2001; Hooper, 2004; Ley et al., 2008; Ruiz et al., 2013). At the genus level, the relative mean abundance of *Alloprevotella* and *UCG-005* in the porcine cecum was elevated by 30.21 and 42.66%, respectively, in the WPS maize addition group compared to the control group. *UCG-005* has fiber-degrading activity, and its abundance in the cecum increased significantly with higher levels of fiber in the diet. *Alloprevotella* is closely related to the digestion of dietary fiber and is abundant in the cecum of the Sujang pig and Jinhua pig (Ren et al., 2020; Zhang et al., 2020). It is the dominant genus, with relative abundance increasing with higher levels of dietary fiber. Consumption of high-insoluble fiber diets during the second trimester of gestation increases the relative abundance of *Alloprevotella* in the feces of sows (Liu, 2019). *Alloprevotella* levels were higher in the feces of grazing Tibetan pigs than in housed Tibetan pigs and Duroc×Landrace×Yorkshire pigs, indicating their higher fiber degradation capacity. Overall, *Alloprevotella* and *UCG-005* may be closely related to crude fiber digestion in Hezuo pigs.

Our correlation analysis of differential genera with SCFAs revealed that the relative abundance of *Bacteroides* and *NK4A214_group* was significantly positively correlated with the amounts of valeric acid and isovaleric acid. They were significantly negatively correlated with propionic acid. Additionally, the relative abundance of *Actinobacillus* was significantly positively correlated with butyric acid. This indicates that feeding WPS maize can influence SCFAs production. This, in turn, affects fiber digestion.

## 5 Conclusion

Adding 10% WPS maize improves fiber digestibility, lowers cecal and colonic chow pH, and increases intestinal cellulase activity and SCFA production in Hezuo pigs. It also enhances intestinal microbiota abundance. These findings provide valuable guidance for the characterization of roughage tolerance in Hezuo pigs.

## Data availability statement

The datasets presented in this study can be found in online repositories. The names of the repository/repositories and accession number(s) can be found in the article/supplementary material.

## Ethics statement

The animal study was approved by Animal Protection and Use Committee of Gansu Agricultural University. The study was conducted in accordance with the local legislation and institutional requirements.

## Author contributions

LW: Data curation, Validation, Visualization, Writing – original draft, Writing – review & editing. PW: Funding acquisition, Project administration, Software, Writing – review & editing. ZY: Conceptualization, Resources, Supervision, Writing – review & editing. PZ: Software, Visualization, Writing – review & editing. XY: Writing – review & editing. RJ: Investigation, Writing – review & editing. YL: Writing – review & editing. JY: Writing – review & editing. SG: Funding acquisition, Project administration, Resources, Writing – review & editing. QY: Conceptualization, Formal analysis, Methodology, Project administration, Resources, Writing – review & editing.
